# Round the Hospitals

**Published:** 1921-09-17

**Authors:** 


### ROUND THE HOSPITALS.

The Treasurer of the London Hospital, Mr. W. T. Paulin, stated at the quarterly meeting last week that, though the financial position is rather less strained, and they expect to get through the year fairly well, he cannot name a time when the closed wards can be re-opened. The gentleman who offered to give the institution £5,000, on condition that three others did the same, has consented to accept a donation of £5,000 from the nurses out of the proceeds of their bazaar as qualifying for this gift, and, as two other £5,000 donors have come forward, the hospital actually benefits through the nurses' exertions to the tune of £20,000. The total raised by the bazaar amounted to £7,000—surely a record amount! The very fact that the nurses of this institution, who are in the best of all positions to gauge the value of the work accomplished, should have undertaken the colossal labour of promoting such an undertaking, in addition to their heavy daily toil, should inspire members of the public to come forward and set the great hospital running once more at full force.

---

The nurses at another London hospital, St. George's, have started a movement for easing the financial situation. St. George's is in debt by over £30,000, and the nursing staff has arranged to have a sale of work in the hospital on October 20 and 21, to which all past "Georgian" nurses are earnestly invited to contribute. They are aiming at £1,000, and experience shows they will get it, for once the impulse has been set going with the proper force it seldom fails to bring in the sum desired. As a token of their earnestness on behalf of their *Alma Mater*, the nurses are taxing themselves at the rate of 2s. 6d. a head, a requisition estimated to bring in £75. Readers are requested to note the date.

---

The new National Provident Scheme which is to come into operation in London on November 1, very closely concerns nurses and especially private nurses. The institutions co-operating in it so far are the London Hospital, St. Thomas's, and the Royal Free. It has nothing to do with National Insurance, and all who come within the income limit, which is fixed at £250 for persons without dependants and £400 to £500 for families, are at liberty to become members. For the payment of a yearly fee of from £1 to £2 members will receive, without further charge, consultations at the hospitals, home nursing, dental treatment, *x*-rays, massage, electrical and radium treatment as required by their case as well as operative and other treatment at the hospitals. It is confidently believed that a very extensive response will be made to this offer, and it cannot fail to be of the utmost service to that large and hard-pushed class of persons whose income is strictly limited. Many nurses will be glad to come under the provisions of the scheme. But there can be no doubt that it will operate still further to limit the sphere of private nursing, already greatly encroached upon by the system of pay beds and pay wards in hospitals. We would urge that the home nursing promised should be undertaken not by hospital institute nurses, but by specially appointed nurses. By this means the extension of the system would mean the extension of good private nursing appointments, and all danger of injuring the nursing profession would be obviated.

Some of the union workhouses in Ireland are to be turned into county hospitals, and the proposal is being made in some quarters to staff them with nuns, a plan not regarded with favour by all trained nurses. The great point in favour of entrusting the management of Irish hospitals and sick wards to a religious order is the popularity of nuns in that country with their patients, which is indisputable. Some of the nursing nuns, though by no means all, have enjoyed the advantage of hospital training, and it ought to be possible to make training a *sine qua non* for the sister-in-charge, and at least a fixed proportion of her staff. Among Anglican nursing orders hospital training is the rule, and no difficulty is experienced in procuring it for such of the sisters as are anxious to qualify seriously for their work. Under modern conditions nursing is no occupation for the amateur, and the religious orders in Ireland should see to it that their ministrations are second to none.

Private nurses will welcome the Dangerous Drugs Act, which came into force on September 1, for many of their perplexities come from the habit of secret self-drugging among their patients. The Act makes it an offence for anyone to have in his possession any preparation containing at least one part in 500 of morphia, or one in 1,000 of heroin, cocaine, or opium, unless the owner is licensed or the drugs are supplied on a doctor's prescription. Paregoric, unless much diluted, and laudanum come within the regulations.

Great satisfaction has been caused among certain Territorial Force nurses by the announcement of the issue of a special War Medal, with ribbon, for nurses not eligible for the 1914 or 1914-15 Star. The conditions of eligibility for this decoration are enrolment in the Force for four years prior to August 4, 1914, and service outside the United Kingdom between the dates August 5, 1914, and November 11, 1918. Application should be made to the Secretary, War Office (T.F. 4), 80 Pall Mall, S.W. 1.

The Scottish Registration Board notifies that the Registrar has received over 500 applications for papers from nurses desirous to register their names, and that a number of nurses have already been accepted. The next few months should enable a fair idea to be formed of the extent to which advantage is likely to be taken of the three Registers in the United Kingdom. The English Registrar should receive at least 5,000 applications to start with, and something like an average of 2,000 a month during the period of grace. We cannot believe, however, that the provisions of the Act have been so far properly put before the nurses of the kingdom. Experience shows more and more the existence of a large number of nurses outside institutions who do not read any nursing paper or take any interest in the government of their profession. How to communicate with these members of the profession and draw them in is the problem which confronts the Registrar.

